# Utilizing Essential Oil Components as Natural Antifungal Preservatives in the Active Packaging of Bread

**DOI:** 10.3390/polym17050697

**Published:** 2025-03-06

**Authors:** Konstantinos Safakas, Georgia C. Lainioti, George Tsiamis, Panagiota Stathopoulou, Athanasios Ladavos

**Affiliations:** 1 Department of Food Science & Technology, University of Patras, GR-30100 Agrinio, Greece; 2 Department of Sustainable Agriculture, University of Patras, GR-30100 Agrinio, Greece; gtsiamis@upatras.gr (G.T.); panstath@upatras.gr (P.S.)

**Keywords:** LDPE, carvacrol, thymol, montmorillonite, oxygen permeability, antioxidant activity, antifungal analysis

## Abstract

The use of essential oil components as natural antifungal preservatives in the active packaging of bread is an innovative approach that leverages the antimicrobial properties of these compounds to extend the shelf life of bread and ensure its safety. The aim of the present work was the thorough investigation of the antioxidant properties and antifungal activity of low-density polyethylene (LDPE or PE) nanocomposite films with organically modified montmorillonite (O) loaded with carvacrol (C) or thymol (T) as a function of time, starting from 2 months and concluding at 12 months. The films PE_OC and PE_OT were prepared through the evaporation/adsorption method, a green methodology developed by our group compatible with food packaging. For a comprehensive analysis of the synthesized films’ oxygen permeability (OTR), measurements were employed, indicating that the incorporation of clay–bioactive nanocarriers into LDPE films reduced their oxygen permeability. A thorough analysis in terms of the antioxidant activity of the films was assessed at various intervals (2, 3, 6, and 12 months), showing high antioxidant activity for films PE_OC10 and PE_OT10 (polyethylene with 10% wt. organically modified montmorillonite loaded with carvacrol or thymol), even at 12 months. Based on the overall analysis, the PE_OC10 film was identified as the most effective option in the antifungal evaluation conducted using white bread, demonstrating substantial inhibition of fungal growth for up to six months.

## 1. Introduction

The demand for safer and more sustainable food preservation methods has significantly increased in recent years, driven by growing consumer preference for natural additives and eco-friendly technologies [1]. This growing interest stems from heightened awareness of the potential health risks associated with synthetic preservatives as well as an increasing commitment to sustainability and reducing environmental footprints [2].

One area of innovation is the use of active packaging systems, which integrate preservation mechanisms directly into packaging materials. Active packaging systems interact with the product and its surrounding environment to enhance safety, extend shelf life, improve hygiene, and maintain the overall quality and sensory attributes of food [3,4,5].

While active packaging is one approach, the broader concept of sustainable food preservation encompasses a variety of methods and technologies aimed at simultaneously extending shelf life, enhancing safety and quality, and minimizing environmental impact. This involves the use of natural additives, biodegradable materials, and energy-efficient processes, thereby aligning with the principles of eco-friendliness and resource conservation while meeting consumer demand for cleaner, safer, and more transparent food preservation solutions [2,6]. These systems represent a significant advancement in food preservation by integrating active components such as antimicrobials, antioxidants, or moisture regulators to combat spoilage and degradation more effectively [7].

The rising demand for chemical-free food has created opportunities for incorporating antimicrobials into the food industry [8]. Antimicrobials represent an innovative technology employed by the food industry to extend food shelf life while addressing critical quality and safety concerns [5]. The incorporation of essential oil components as natural antifungal agents holds great promise for enhancing food safety and quality [9]. Essential oils (EOs), which are complex mixtures of volatile compounds derived from plants, are well-known for their antimicrobial and antifungal properties. These benefits stem from their bioactive components, such as terpenes, phenols, and aldehydes. The primary components of essential oils are categorized into monoterpenes, monoterpenoids, and phenylpropanoids, featuring alcohol or aldehyde functional groups [10]. Essential oils are recognized as safe (GRAS) by the U.S. Food and Drug Administration (FDA) that are permitted for use in food for human consumption [11]. Their antimicrobial properties against foodborne pathogens, along with their function as food flavorings, make them highly suitable for use in food packaging applications. Their use has been evaluated using different techniques, such as (a) the exposure of the product to EO vapors prior to their packaging; (b) their incorporation in sachets to be inserted in the packaging; and (c) their incorporation in packaging films of different compositions [12].

In recent years, there has been increasing interest in integrating essential oils into food packaging systems, particularly those containing clay-based components, due to their potential to act as natural antimicrobial agents. Clays, as abundant natural materials, have long been used in various industries for their adsorbent and barrier properties. When combined with essential oils, clays not only retain their functionality but also enhance the material’s ability to inhibit microbial growth. This synergistic approach offers a promising solution to extend the shelf life of food products while reducing the need for synthetic preservatives. Furthermore, the incorporation of essential oil components into clays can lead to the development of active packaging systems that control the release of antimicrobial agents directly onto the foods’ surface, providing targeted protection against contamination [9].

Bread and bakery products are the main products consumed around the world and recently the extension of the supply chain has been remarkable. Increasing bread consumption was reported as a 3.66% Compound Annual Growth Rate (CAGR) between 2020 and 2030 in the global market, attributed to its convenience, nutritional value, and appealing taste [13]. The resulting need to extend the shelf life of baked goods is a major challenge for the bakery industry [14]. The major limiting factor in the shelf life of such intermediates is their susceptibility to microbial infections [14]. In particular, the development of molds in bread causes significant food waste and constitutes an important economic problem, while being one of the primary concerns of consumers. In Europe, economic losses related to the presence of fungi in bread have been estimated at more than ₤200 million per year [15]. For instance, bread waste alone in Europe is estimated to account for up to 4% of total food waste, with fungi being one of the key contributors to spoilage. Fungal spores originate mainly from the initial materials and facilities’ air, contributing to the off-flavor, allergic compounds, and mycotoxins [16,17]. Bread and bakery products can spoil rapidly, mainly due to the growth of *Penicillia* and *Aspergilli* species. *Penicillium* spp. are able to grow in bread surfaces and form a greenish-blue layer [17]. To prevent spoilage, the main alternative is the use of preservatives. However, this method can become less effective due to the resistance of some fungal strains and is often poorly accepted by consumers. Recent studies have explored various methods to integrate essential oils into bread packaging materials. Císarová et al. [18] investigated the in vitro and in situ effects of selected essential oils in vapor phase against bread spoilage of *Aspergillus* spp. The results demonstrated that thyme, clove, oregano, cinnamon, and lemongrass were highly effective in inhibiting the tested toxigenic *Aspergillus* species in the vapor phase, suggesting their potential as natural preservatives in bread packaging. In another work, Noshirvani et al. [19] developed stable film-forming dispersions with the use of carboxymethyl cellulose, chitosan, and oleic acid added with cinnamon or ginger essential oils. Experiments conducted on sliced soft bread showed that ginger-based films extended the shelf life by up to 20 days compared to the control. Even more promising, cinnamon-based films completely inhibited fungal growth on bread slices, with this effect lasting for more than 60 days. Sripahco et al. [20] made biodegradable films incorporating Anethum graveolens (dill) essential oil which showed enhanced mechanical strength and effective inhibition of *Aspergillus* growth on bread during a three-week storage period.

The aim of this study was to investigate the antioxidant properties and antifungal activity of low-density polyethylene films with organically modified montmorillonite loaded with carvacrol or thymol over a 12-month period. The films were prepared through a sustainable approach developed in our laboratory that directly incorporates essential oil components into clays using the evaporation/adsorption method, eliminating the need for organic solvents, which are harmful not only for the environment but also for human health. This makes the method an eco-friendly alternative approach compared to traditional methods that use solvent-based processes.

The research focused on evaluating the effectiveness of these films as natural antifungal preservatives in active bread packaging. This study assessed the films’ oxygen permeability, antioxidant activity at various intervals (2, 3, 6, and 12 months), and their ability to inhibit fungal growth on bread. The ultimate goal was to identify the most effective film for enhancing both the shelf life and safety of bread. While many studies focus on the antimicrobial or antioxidant properties of essential oils, this study specifically emphasizes the antifungal activity, showing how these films can prevent fungal growth in bread for up to six months (180 days)—an important aspect of food spoilage prevention.

## 2. Materials and Methods

### 2.1. Materials

Carvacrol (5-isopropyl-2-methylphenol) 98%, thymol (2-isopropyl-5-methylphenol) ≥98.5%, and DPPH (2,2-diphenyl-1-picrylhydrazyl) were purchased from Sigma-Aldrich (Aldrich, Steinheim, Germany) and used without further modification. The organically modified montmorillonite Nanomer^®^ I.44P, a surface-modified montmorillonite clay with 40% wt. dimethyl dialkyl (C14–C18) amine, was produced by Nanocor Inc. (Hoffman Estates, IL, USA), supplied by Sigma-Aldrich (St. Louis, MO, USA), and used as received without any further treatment. The solvent ethanol absolute was purchased from Merck (Merck KGaA, Darmstadt, Germany). Low-density polyethylene (DOW^TM^ LDPE 352E) was kindly provided from Achaika Plastics S.A. (Aigio, Greece).

### 2.2. Bioactive Nanocarriers

The preparation of the bioactive nanocarriers has been mentioned in detail elsewhere [21]. The incorporation of carvacrol (C) and thymol (T), main components of oregano and thyme essential oil, respectively, into the organically modified montmorillonite (O) was conducted using the evaporation/adsorption method, an eco-friendly technique developed by our team that is suitable for food packaging. In brief, the appropriate quantity of the bioactive compound was placed on a glass plate at the bottom of a closed and heated chamber. Next, a glass plate containing 3 g of clay was positioned above the bioactive compound. The chamber was sealed and maintained at 120 °C for 96 h, allowing intra-layer and surface adsorption of the bioactive compounds on O.

### 2.3. Preparation of LDPE/Clay Bioactive Nanocomposite Film

LDPE and OT or OC were melt-compounded using a Haake Mini Lab II twin co-rotating extruder (ThermoScientific, ANTISEL S.A., Athens, Greece). The process was conducted at 160 °C with a screw speed of 100 rpm for a total of 10 min. The resulting masterbatch was then pressed using a Manual Hydraulic Press with heated platens (Specac, ANTISEL S.A., Athens, Greece) between two Teflon sheets at 120 °C and 2 tons of pressure for 2 min, followed by rapid quenching in an ice-water bath to form the film.

### 2.4. Oxygen Permeability Measurement

An O.P.A. 8001 oxygen permeability analyzer (Systech Illinois Instruments Co., Johnsburg, IL, USA) was used to test the oxygen permeability of the membranes. The experimental procedure was carried out according to ASTM D3985 [22] (23 °C and RH = 0%), and then the oxygen permeability coefficient (PeO_2_) for each membrane was calculated according to the following equation:

PeO_2_ = OTR × ∆x (1)

where OTR is the oxygen transmission rate through the film (cm^3^ O_2_ STP·cm^−2^ film area·s^−1^) and ∆x the mean film thickness (cm). A low value of the permeability coefficient indicates a high barrier capacity.

### 2.5. Determination of Antioxidant Activity Based on the Free Radical Binding Capacity of DPPH

The antioxidant activity of the samples was determined using the DPPH (2,2-diphenyl-1-picrylhydrazyl) free-radical scavenging method. This method monitors the bleaching rate of the stable free radical DPPH• at a characteristic wavelength in the presence of the sample [23,24,25]. Initially, ethanolic solutions of the sample (1 mg/mL) and DPPH (1 mM) were prepared. Appropriate volumes of ethanol, the ethanolic sample solution, and 400 μL of the EtOH-DPPH solution were combined to a final volume of 4 mL. The samples were kept at room temperature (RT) in the dark for 20 h and the absorbance at 517 nm was then recorded. The control solution, consisting of ethanol and DPPH, was also measured at 517 nm. All measurements were conducted in triplicate and the results are presented as the mean average.

The antioxidant capacity was assessed using the IC50 parameter, which indicates the concentration of antioxidant required to reduce the initial concentration of the compound by 50%. A lower IC50 value signifies a stronger antioxidant effect.

### 2.6. Bread Packaging and Assessment of Its Quality

Two kinds of white bread, sliced bread (cut into four triangles) and baguette, were assessed during storage by visual inspection of the fungal growth. One slice of baguette and one triangle of the sliced bread were stored in clear plastic polyethylene terephthalate (PET) punnets with or without (control) the prepared PEOC10 film. Two films were used for each test; one was placed at the bottom of the bread and the other at the upper side. Neat LDPE films were also used as a control. The experiments were conducted over 180 days at room temperature (25 °C) with a relative humidity of 65% and their visual appearance was observed during a period of three months.

### 2.7. Antimicrobial Analysis of Bread Samples

The bread samples (sliced bread and baguette) were stored at room temperature (25 ± 2 °C) and observed for 6 months. Two PEOC10 membranes were used; one was placed at the bottom of the bread and the other at the upper side. Visual observations for microbial (especially mold) growth were carried out on the bread samples.

To make the stock solution for each sample, a small piece of each sample was aseptically weighed and suspended in phosphate buffered saline (PBS), homogenized in a commercial blender for 1 min, and mixed using a shaker incubator at 160 rpm for 40 min. The material was analyzed using a 10-fold dilution and serial dilutions were prepared up to 10^−7^.

Then, 0.1 mL of each dilution was spread evenly on the LB medium (Luria–Bertani), a commonly used and nutritionally rich medium for culturing bacteria, and incubated. Plates were screened for the presence of discrete colonies after the incubation period (30 °C for 48 h) and the actual numbers of bacteria were estimated as colony forming units per gram (CFUs/g).

In the case of the TFC (total fungal count), potato dextrose agar (PDA) was inoculated with 0.1 mL of different dilutions of each sample and thereafter the inoculated plates were incubated at 28 ± 2 °C for 5 days. Upon incubation, the total number of colonies appearing on the plates was counted manually and the number was multiplied by the dilution. The TFC per g of the samples was recorded as CFUs/g of bread. All tests were performed at least in triplicate and the results are presented as the mean average.

### 2.8. Statistical Analysis

The data obtained from oxygen permeability, antioxidant activity, and mold enumeration were expressed as mean ± standard deviation (SD) and all analyses were performed in triplicate. Data were analyzed using one-way analysis of variance (ANOVA).

## 3. Results

### 3.1. Preparation of LDPE Films Loaded with Bioactive Nanocarriers

In this study, the bioactive nanocarriers were incorporated into the LDPE matrix using a mini twin-screw extruder to create masterbatches, which were then formed into films. The selected bioactive nanocarriers were organically modified clay loaded with carvacrol and thymol (with a 1:1 nominal ratio of bioactive compounds to clay), as reported in detail elsewhere [21]. Table 1 outlines the compositions of all blends used to prepare films from LDPE containing O loaded with the bioactive compounds C and T.

### 3.2. Membrane Oxygen Permeability Measurements

The detrimental impact of oxygen on the quality of various food products is well-established in the literature [26,27,28]. It contributes to lipid oxidation, microbial growth, enzymatic browning, and vitamin degradation. Therefore, developing packaging materials that can minimize or slow down food oxidation is essential for preserving quality and extending shelf life. Table 2 presents the mean oxygen transmission rate (OTR) values for all tested LDPE films, including pure LDPE film for reference.

Using these OTR values and the film thickness, the oxygen permeability (PeO₂) values were calculated and are also included in Table 2 for comparison. As shown by the calculated oxygen permeability (PeO_2_) values in Table 2, the oxygen permeability of pure LDPE was 2.06 ± 0.12 × 10^−8^ cm^2^/s. Films containing bioactive compounds and clay nanohybrids demonstrated improved oxygen barrier properties compared to neat PE film, ranging between 1.52 ± 0.09–1.68 ± 0.13 × 10^−8^ cm^2^/s. Incorporating MMT and carvacrol or thymol into the LDPE matrix enhanced the oxygen barrier properties compared to neat LDPE. This improvement is attributed to the MMT layers acting as impermeable barriers in the diffusion path of oxygen molecules. As a result, the enhanced barrier properties can be attributed to a reduction in the free volume fraction, as a portion of the permeable polymer replaced by impermeable MMT layers. This decrease in permeability is due to the delamination and dispersion of impermeable clay platelets, which act as barriers in the diffusion pathway. These barriers compel the diffusing molecules to follow a more tortuous path, thereby slowing down and reducing gas molecule diffusion [29,30]. Furthermore, these findings suggest that during the extrusion process, carvacrol and thymol were solubilized and may have migrated to the amorphous regions of the polymeric structure due to their oily characteristics. Recent studies have investigated the incorporation of oregano and thyme essential oils into low-density polyethylene (LDPE) films to enhance their oxygen barrier properties. For instance, Coelho et al. [31] developed active LDPE films with varying concentrations of oregano essential oil (1–6% *w*/*w*) and observed a decrease in carbon dioxide permeability, suggesting improved barrier properties. Similarly, Tornuk et al. [32] indicated that the inclusion of nanoclays, such as montmorillonite (MMT) and halloysite (HNT), as well as thymol and carvacrol, significantly improved the oxygen barrier properties of LLDPE films.

### 3.3. Antioxidant Activity

**Figure 1:**
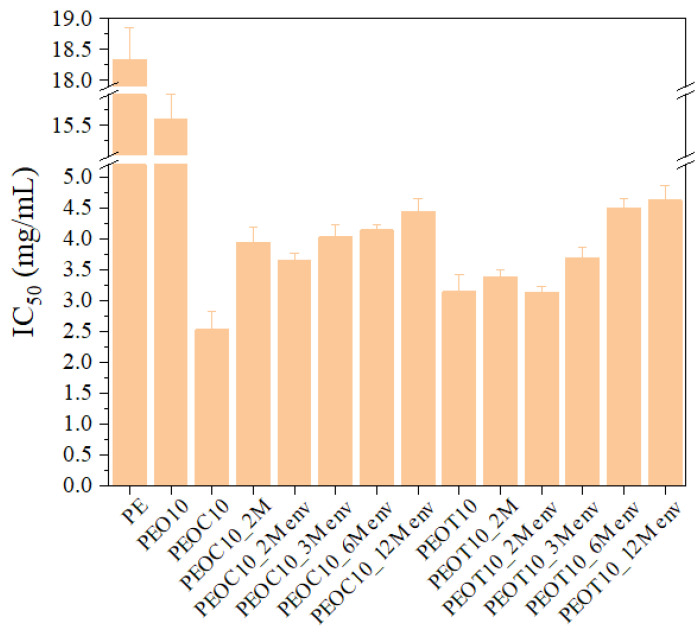
Antioxidant activity (IC50) of PE films containing organically modified clay nanocarriers (O) infused with carvacrol (C) and thymol (T) for a period of 2, 3, 6, and 12 months.

Antioxidant activity in food packaging is essential for maintaining food safety. Studies suggest that various factors, including temperature, light exposure, and the type of food simulant, can impact the effectiveness of antioxidant properties in packaging materials [33]. The antioxidant activity of PE films was evaluated using the DPPH assay, a widely used method for assessing the free radical scavenging properties of various plant-derived compounds [34]. The antioxidant power was determined using the IC50 parameter, which denotes the concentration of antioxidant required to reduce the compound’s initial concentration by 50%. Figure 1 presents the antioxidant activity of all obtained PE/OC and PE/OT active films over 2, 3, 6, and 12 months (M).

The experiments for 2M were conducted in both a sealed bag and an open environment. Since no significant difference appeared in the IC50 values between the two environments, the remaining experiments for 3M, 6M, and 12M were conducted in an open environment to simulate real industrial conditions. As shown in Figure 1, the antioxidant activity, measured as the IC50 value—the concentration of the active films required to inhibit 50% of DPPH free radicals—ranged from 2.5 ± 0.29 mg/mL to 4.4 ± 0.2 mg/mL for PEOC and from 3.1 ± 0.27 to 4.6 ± 0.24 mg/mL for PEOT. Even after 12 months, the IC50 value for PEOC and PEOT films remained between 4.4 ± 0.2 and 4.6 ± 0.2 mg/mL, which is significantly lower than the IC50 values of neat PE and PEO10 films, recorded at 18.3 ± 0.51 mg/mL and 15.6 ± 0.4 mg/mL, respectively.

Table 3 presents the variation in antioxidant activity of different PEOC and PEOT samples over time (2M, 3M, 6M, and 12M) and under various conditions (sealed bag and open environment) relative to the PE sample. As may be observed, both PEOC10 and PEOT10 show strong antioxidant activity relative to PE, with values ranging from +74% to +86% depending on the sample. Over time (from 2M to 12M), antioxidant activity seems to decrease slightly, which may indicate some form of degradation or a loss of effectiveness of the antioxidants in the formulations. Environmental factors appear to have a small impact on the antioxidant activity, with some samples showing slightly lower values when exposed to environmental conditions (env). However, the difference is not remarkable in most cases.

### 3.4. Assessment of Food Preservation

The prepared PEOC10 film was used for the preservation of white bread which was purchased from a local bakery to assess its potential ability for food preservation. The choice of white bread was based on its high susceptibility to spoilage, especially mold growth, making it an appropriate model for evaluating how well packaging can maintain freshness. Moreover, the high moisture content of white bread makes it very susceptible to fluctuations in humidity and moisture levels.

**Figure 2:**
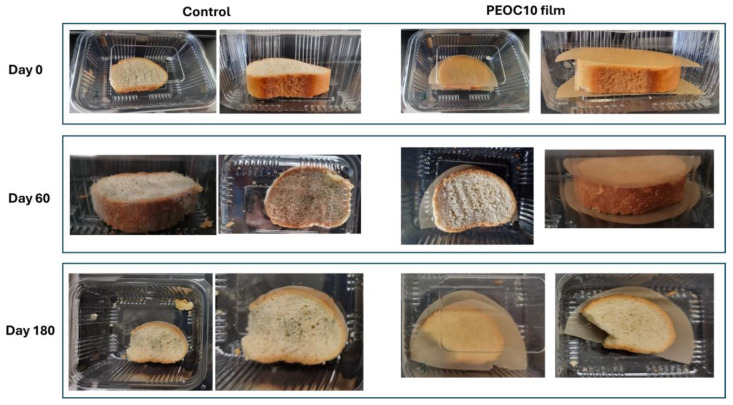
Mold growth in white baguette bread sealed in polyethylene package with the PEOC10 film or without film (control).

**Figure 3:**
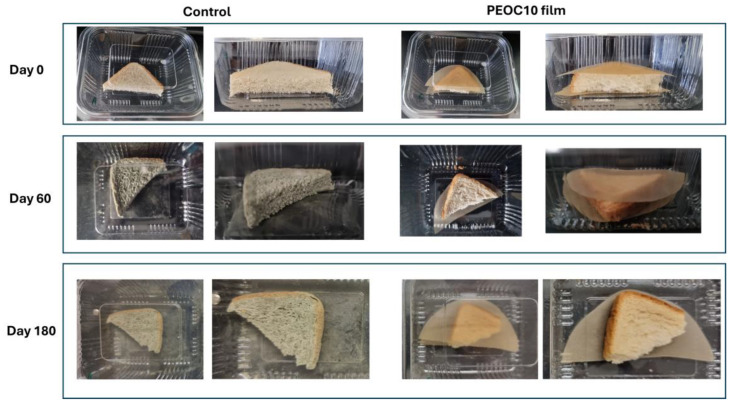
Mold growth in white sliced bread sealed in a polyethylene package with the PEOC10 film or without film (control).

For the testing, one triangle of sliced bread and one slice of baguette were sealed in a commercial plastic package (polyethylene) with or without (control) the prepared PEOC10 film. The visual appearance of the samples was observed during a period of six months. Table 4 shows the fungal growth intensity of the two bread samples during the assessment of visual observation. Figure 2 and Figure 3 depict photographs of the two kinds of bread obtained at the beginning of the experiment (Day 0), 2 months (Day 60), and 6 months (Day 180).

As may be observed in Figure 3, the sliced bread exhibited mild growth of fungi after 60 days.

### 3.5. Evaluation of Fungal Growth

**Figure 4:**
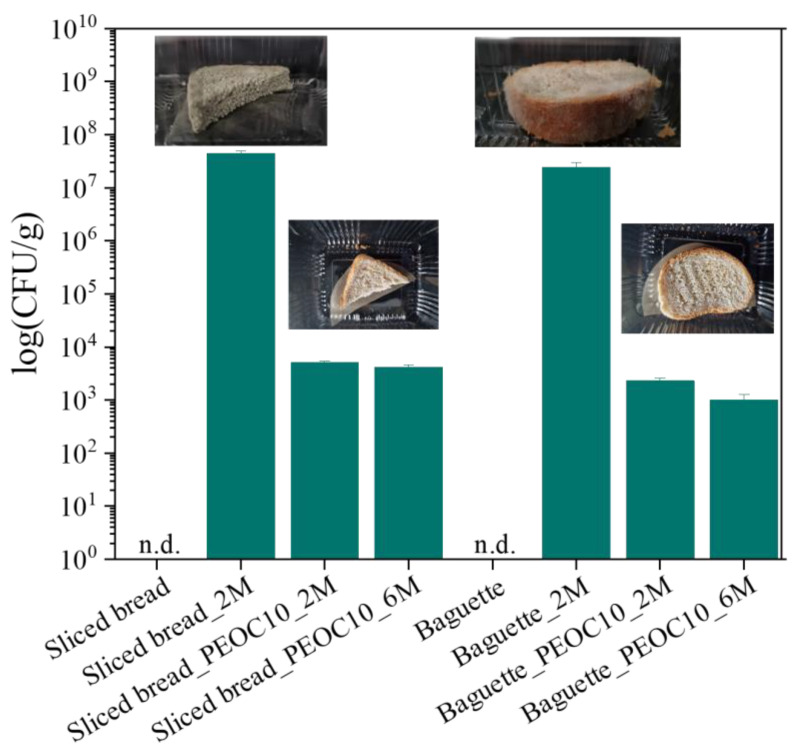
Number of mold growth on sliced bread and baguette during storage for 0 days, 60 days (2M), and 180 days (6M) at 25 °C and 65% RH.

Microbial spoilage, especially fungal spoilage, is the main cause of the significant waste in packaged bakery products. Therefore, microbiological analysis was performed on the bread samples (sliced bread and baguette) with or without the membranes (PEOC10) during their storage at room temperature (25 ± 2 °C) and observation for 180 days. Regardless of the type of bread, no bacterial growth was observed on LB plates during the entire process, so the main focus became the mold growth. The results of mold enumeration are presented in Figure 4.

Bread samples without membranes presented the same behavior and showed high fungal counts up to 10^7^ CFU/g after 60 days of storage. These bread samples showed signs of spoilage within the first ten days of storage. From those days, the infections increased with time, covering almost the whole surface of the bread on day 180. Bread covering by active membranes (PEOC10) proved very effective in retarding fungal development and the bread samples were found to harbor fungi within the acceptable microbial limits (ranging between 1–3 × 10^3^ CFU/g) even on the 180th day of storage (Figure 4). In the case of membrane-covered breads, the fungal growth was successfully inhibited even during prolonged storage.

Several authors have reported that the use of active packaging or active materials can reduce the incidence of mold infections in bread. In these studies, oregano essential oil or cinnamon essential oil were used at high concentrations, and some detrimental effects on sensory properties were also reported owing to the intense aroma of these substances [35,36]. Carvacrol is a phenolic compound found in oregano oil and is known for its antimicrobial properties. Because of these properties, it is being explored for use in active packaging, which involves packaging materials that can interact with the food or environment to extend shelf life, preserve quality, and improve food safety. Carvacrol is effective at inhibiting fungal growth, which is a primary cause of mold in bread. By embedding carvacrol in the packaging material, the active compound could continuously interact with the bread’s surface, reducing the likelihood of mold growth over time [37]. This compound is considered safe and has been classified as GRAS by the American Food and Drug Administration [38]. The antifungal activity of carvacrol has been previously demonstrated in several studies [39,40,41,42,43]. However, the use of polyethylene (PE) films with organically modified montmorillonite (O) as a carrier system for the release of carvacrol with an antifungal function in baked goods has not yet been evaluated.

Baked goods have a sterile surface after the baking process and mold spores are inactivated during baking [44]. Additionally, in the present study, in both samples, regardless of the type of bread, on day 0, there was no reported contamination (Figure 4). In bakery products like bread, the acceptable level of mold would generally be zero or very low by the time the product reaches the consumer. Further packaging and slicing procedures, however, can quickly lead to microbial contamination of products. Air, as well as contact with surfaces and equipment, is also often a source of contamination [14]. Bread typically has a short shelf life and the development of mold is one of the primary factors that limits this. Mold growth is usually expected to occur within a few days to a week, depending on storage conditions, humidity, and the presence of preservatives or active packaging. In fresh unsealed bread (like the baguette), fungi will typically begin growing on the surface of the bread, especially in areas that are exposed to air. Mold prefers the soft, moist outer crust and the crumb (the inside of the bread). The common species are *Penicillium* (green/blue), *Aspergillus* (green/black), and *Rhizopus* (white fuzz turning black). Fungi often form as a patchy layer on the crust or on exposed parts of the bread. The sliced bread is exposed inside when sliced and the moist crumb of the bread is exposed to the air, which can lead to fungal growth more quickly. The fungus may spread from the edges of the slice inward. The common species are *Penicillium*, *Aspergillus*, *Rhizopus*, and *Mucor* (white fluffy mold). In the present study, fungal growth was observed in bread samples on day 60 (2 months), ranging from 2.3 × 10^7^ cfu/g to 4.4 × 10^7^ cfu/g (baguette and sliced bread, respectively). On the other hand, the bread samples covered with carvacrol-incorporated packaging films revealed no more than 5 × 10^3^ cfu/g even after 180 days (6 months). LDPE–clay nanocomposite films containing carvacrol effectively inhibited the microbial growth of fungi for more than 180 days. The results showed a noticeable reduction in mold growth and the bread maintained a fresher appearance for a longer period. In our study, the bread was stored in plastic containers without airflow, and this is more likely to develop mold as spores cannot escape and moisture accumulates. Additionally, the storage temperature was around 20–25 °C, conditions in which fungi can grow optimally. Although the storage conditions promoted fungal growth, the carvacrol-incorporated packaging films revealed significant fungal inhibition. Additionally, the individual slices of bread were just wrapped in films and this application process could be optimized and lead to better results. Moreover, the combination of carvacrol’s antifungal properties and LDPE’s moisture retention helps prevent both mold and the drying out of bakery products, addressing two common issues with bread storage.

It is worth noting that the European Commission Regulation (EC) No. 2073/2005 sets microbiological criteria for food safety, including those for molds and yeasts. While the regulation does not give an exact limit for mold counts in bakery products, it does establish limits for yeast and molds in foods. For ready-to-eat bakery products, yeast and mold counts should typically be below 10^2^ CFU/g to meet food safety standards. In our study, after 60 days of storage, mold counts were lower than 5 × 10^3^ cfu/g, compared with the greater than 2.3 × 10^7^ cfu/g counts found in the control group. Even after 180 days of bread storage with PE_OC10, the mold counts remained close to the allowable limits for baked goods.

To present a wider valuation on the antifungal activity of carvacrol and thymol essential oil components used in the current work, Table 5 below compares the physicochemical properties, antioxidant properties, and antifungal activity of these two EOs commonly used in food packaging applications.

It is worth noting that the results of antioxidant and antifungal activities are in strong accordance, demonstrating the effectiveness of active membranes in both inhibiting oxidative degradation and preventing fungal growth. The sustained antioxidant activity of PEOC and PEOT films, with IC50 values significantly lower than those of neat PE and PEO10 even after 12 months, indicates long-term stability and effectiveness in scavenging free radicals. This aligns with the antifungal performance, where the active membrane PEOC10 effectively inhibited fungal growth on bread samples, maintaining microbial counts within acceptable limits even after 180 days of storage. The correlation between the antioxidant and antifungal effects suggests that the active compounds in the membranes contribute to both oxidative stability and microbial resistance, reinforcing their potential as multifunctional packaging materials for extended food preservation.

This dual functionality highlights the versatility of active compounds in packaging applications over periods as long as 6 months, which has not been extensively explored in existing research. A recent study investigated the use of carvacrol-loaded LDPE films and found that they had the capacity to inhibit fungi by 99% compared to neat LDPE [48]. Further, there are other studies examining the combination of carvacrol and thymol in a 50:50 ratio for active food packaging. The association of carvacrol and thymol produced an in vivo (strawberry) synergistic antimicrobial effect against gray mold. Several studies have reported the incorporation of carvacrol and thymol into various materials such as polylactic acid, polycaprolactone, polypropylene, or polyethylene [49,50].

Moreover, Shemesh et al. [51] studied halloysite (HNT)/carvacrol hybrids which were melt-compounded with LDPE. The LDPE/(HNT/carvacrol hybrid) films showed very good antibacterial and antibiofilm properties against *E. coli* and *L. innocua*, as well as antifungal activity against A. alternata in vitro and against a variety of other fungi involved in food spoilage. Devecioglu et al. [52] studied the in vivo antifungal activity of clove and cinnamon nanofiber films produced with 6% poly(vinyl alcohol), 2% β-cyclodextrin, and 2% EO. The visible fungal growth was tested for a 6-day period for packed and nonpacked bread samples stored under sterile conditions at 25 °C. Sliced bread packed with either clove EO or cinnamon EO nanofiber films showed no sign of any fungal growth.

Fernandes et al. [53] developed active films with cellulose acetate and 0.5%, 1.5%, 2.5%, and 3.5% oregano essential oil in order to examine the antifungal effectiveness against fungal spoilage on hamburger buns’ shelf life. The results showed delayed growth of filamentous fungi on hamburger buns until the 29th day. Suwanamornlert et al. [47] confirmed a delay in the visible growth of yeast and mold in bread by 7 and 9 days, respectively packed with poly(lactic acid)/poly(butylene-succinate-co-adipate) (PLA/PBSA) blend films containing 3 and 6% wt. of thymol. In another work [46], poly(lactic acid) and poly(butylene adipate terephthalate) (PBAT/PLA) films contained 2 and 5% carvacrol caused a prevention of mold growth and an extension of shelf life of bread and butter cake by 2–4 days. Moreover, Heras-Mozos et al. [54] prepared a polyethylene (PE) aqueous emulsion and ethylene–vinyl alcohol copolymer (EVOH) and zein hydroalcoholic solutions with 0.25 and 0.5% of garlic extract and bread aroma for the coating of PE films. The results indicated that PE film coated with zein containing 0.5% garlic extract and bread aroma maintained bread free of mold infection for 30 days. From the above-mentioned references, we observe that there are a lot of studies reported in the literature that have explored essential oils and antimicrobial packaging for short-term applications. Our study emphasizes the long-term efficacy of films over a period of 12 months which is a significantly longer period compared to the existing literature. Sensory evaluation was also conducted by three researchers in the laboratory. The aroma and taste intensity of the control bread (without film), bread packaged with the active PEOC10 film, and the active PEOC10 film itself were compared. As far as the aroma of bread was concerned, on a 1–5 scale, control bread would score 1, the active PEOC10 film would score 5, and bread packaged with the active PEOC10 film would score 2. Regarding the taste intensity of bread, two researchers who tasted the bread packaged with the active PEOC10 film did not realize any changes in taste, whereas one researcher noticed a slight oregano flavor in the bread.

The results of this study highlight that packaging is a feasible option to reduce fungal growth and mycotoxin production. The incorporation of carvacrol into LDPE films can kill or extend the lag phase or decrease mycelial growth and spore germination during the stationary phase of mold growth. Additionally, the controlled release of carvacrol from the packaging helps prevent microbial contamination without negatively affecting the food’s sensory characteristics (taste, smell, etc.). This extended period of study provides valuable insights into the viability and long-term effectiveness of films intended to be used in food packaging. Above all, it is important and innovative to understand how these films retain their antioxidant and antifungal activity for up to 12 months, potentially making them an innovative solution in food packaging.

## 4. Conclusions

The present work provides a detailed assessment of the films including antioxidant activity, antifungal effectiveness, and oxygen permeability. This comprehensive analysis is valuable for understanding the overall performance of these films as active packaging materials. The incorporation of carvacrol and thymol, essential oil components with known antimicrobial properties, into low-density polyethylene (LDPE) films for food preservation is a relatively innovative approach. These natural bioactive compounds offer a green alternative to synthetic preservatives, which is increasingly sought after for sustainable food packaging solutions.

The present study demonstrates that the incorporation of organically modified montmorillonite loaded with carvacrol (OC) and thymol (OT), essential oil components, into LDPE films significantly enhances their antioxidant properties. These bioactive compounds offer a natural method for improving the preservation of bread. The films PE_OC10 and PE_OT10 exhibited high antioxidant activity, showing sustained effectiveness even after 12 months. This indicates that these films can provide long-term protection against oxidative degradation in packaged food. The inclusion of clay–bioactive nanocarriers into LDPE films led to a reduction in oxygen permeability, which could further enhance the shelf life and freshness of bread by limiting the exposure to oxygen, a key factor in spoilage.

The PE_OC10 film demonstrated significant antifungal activity, inhibiting fungal growth on white bread for up to six months. This makes PE_OC10 the most effective option for antifungal preservation in the study. The green methodology employed in preparing these films is environmentally friendly and aligns with the increasing demand for sustainable food packaging solutions that not only preserve food but also ensure its safety without the need for synthetic preservatives. The strong correlation between antioxidant and antifungal activities confirms the effectiveness of active membranes in extending shelf life by simultaneously preventing oxidative degradation and inhibiting fungal growth, making them promising candidates for sustainable food packaging.

It is worth noting that studies reported in the literature refer to antifungal packaging materials with shorter periods of efficacy. The fact that PE_OC10 films were able to prevent fungal growth for up to 6 months in bread is an innovative finding of the present work. This -long-term antifungal effectiveness is remarkably significant for bread, which is prone to mold growth. The ability for shelf-life extension in combination with the preservation of freshness and safety is an important outcome that differentiates our work from existing studies.

Although the present study provides promising results, there are quite a few paths for future investigation into the optimization of these active films’ performance. Further investigation into the optimization of film composition, or the use of other bioactive compounds, could lead to the improvement of these films’ efficacy. In addition, studying the release kinetics of these bioactive agents over time would give valuable information in the understanding of their controlled release mechanisms and as well as the way they affect food preservation over extended periods. Sensory evaluations of bread packaged with these films will be also of utmost importance to evaluate how they affect taste, odor, aroma, and general acceptability to consumers. Finally, further studies may be conducted in order to test all the required parameters of these films for large-scale commercial applications. These future investigations will support the improvement of the current method and contribute to the development of sustainable packaging solutions for the food industry.

## Figures and Tables

**Table 1:** Blend compositions for LDPE films.

Membrane	Blends	Nominal Clay: Bioactive Substance Ratio (r)	Composition (% wt.)
PE	LDPE	-	100
PE_O10	LDPE/O	-	90/10
PE_OC5	LDPE/OC	1:1	95/5
PE_OC10	LDPE/OC	1:1	90/10
PE_OC20	LDPE/OC	1:1	80/20
PE_OΤ5	LDPE/OT	1:1	95/5
PE_OΤ10	LDPE/OT	1:1	90/10
PE_OΤ20	LDPE/OT	1:1	80/20

**Table 2:** OTR and PeO_2_ values for films loaded with bioactive nanocarriers as well as pure polyethylene film.

Membrane	Film Thickness (mm)	OTR (mL·m^−2^·Day^−1^)	PeO_2_ (10^−8^ cm^2^/s)
PE	0.1404	1268.18	2.06 ± 0.12
PEOC5	0.1025	1391.25	1.64 ± 0.02
PEOC10	0.1075	1285.5	1.57 ± 0.12
PEOC20	0.1243	1172.92	1.68 ± 0.13
PEOT5	0.1058	1260.33	1.54 ± 0.11
PEOT10	0.1021	1337.06	1.56 ± 0.22
PEOT20	0.1058	1246.58	1.52 ± 0.09

**Table 3:** Variation in antioxidant activity relative to PE.

Membrane	Increase (%)
PEOC10	+86
PEOC10 2M	+79
PEOC10 2M env	+80
PEOC10 3M env	+78
PEOC10 6M env	+77
PEOC10 12M env	+76
PEOT10	+83
PEOT10 2M	+82
PEOT10 2M env	+83
PEOT10 3M env	+80
PEOT10 6M env	+76
PEOT10 12M env	+74

**Table 4:** Assessment of visual observation of bread samples stored for 180 days at 25 °C.

Bread Sample		Fungal Growth Intensity	
	Day 0	Day 60	Day 180
Sliced Bread	−	++	+++
Sliced Bread_PEO10C	−	−	−
Baguette	−	+	++
Baguette_PEO10C	−	−	−

Note: − absence of fungal growth, + fungal growth 25% of surface, ++ fungal growth 25–50% of surface, and +++ fungal growth >75% of surface.

**Table 5:** Physicochemical properties, antioxidant capacity, and antifungal activity of carvacrol and thymol essential oil components used in the present study.

Essential Oil Component	Physicochemical Properties	Antioxidant Properties	Antifungal Activity (cfu/g)	References
Carvacrol	Molecular weight: 150.22 g/mol, Boiling point: 238 °C, Density: 0.979 g/cm^3^	DPPH assay: IC50 = 79.75 µg/mL [45]	Reduction in *Penicillium* spp. and *Rhizopus* spp. growth in bread packaging	Klinmalai et al. [46]
Thymol	Molecular weight: 150.22 g/mol, Boiling point: 232 °C, Density: 0.876 g/cm^3^	DPPH assay: IC50 = 70.06 µg/mL [45]	Reduction in *Aspergillus* spp. and *Penicillium* spp. growth in bread packaging	Suwanamornlert et al. [47]

## Data Availability

The original contributions presented in this study are included in the article. Further inquiries can be directed to the corresponding authors.

## References

[1] García-Díez J., Gonçalves C., Grispoldi L., Cenci-Goga B., Saraiva C. (2021). Determining food stability to achieve food security. Sustainability.

[2] Lisboa H.M., Pasquali M.B., dos Anjos A.I., Sarinho A.M., de Melo E.D., Andrade R., Batista L., Lima J., Diniz Y., Barros A. (2024). Innovative and Sustainable Food Preservation Techniques: Enhancing Food Quality, Safety, and Environmental Sustainability. Sustainability.

[3] Kadirvel V., Palanisamy Y., Ganesan N.D. (2024). Active Packaging System—An Overview of Recent Advances for Enhanced Food Quality and Safety. Packag. Technol. Sci..

[4] Kumar K.V.P., Suneetha J., Kumari B.A. (2018). Active packaging systems in food packaging for enhanced shelf life. J. Pharmacogn. Phytochem..

[5] Fadiji T., Rashvand M., Daramola M.O., Iwarere S.A. (2023). A Review on Antimicrobial Packaging for Extending the Shelf Life of Food. Processes.

[6] Hussain S., Akhter R., Maktedar S.S. (2024). Advancements in sustainable food packaging: From eco-friendly materials to innovative technologies. Sustain. Food. Technol..

[7] Zhang H., Tikekar R.V., Ding Q., Gilbert A.R., Wimsatt S.T. (2020). Inactivation of foodborne pathogens by the synergistic combinations of food processing technologies and food-grade compounds. Compr. Rev. Food Sci. Food Saf..

[8] Arshad M.S., Batool S.A. (2017). Natural antimicrobials, their sources and food safety. Food Additives.

[9] Tomić A., Šovljanski O., Erceg T. (2023). Insight on Incorporation of Essential Oils as Antimicrobial Substances in Biopolymer-Based Active Packaging. Antibiotics.

[10] de Sousa D.P., Damasceno R.O.S., Amorati R., Elshabrawy H.A., de Castro R.D., Bezerra D.P., Nunes V.R.V., Gomes R.C., Lima T.C. (2023). Essential Oils: Chemistry and Pharmacological Activities. Biomolecules.

[11] Da Silva Dannenberg G., Funck G.D., dos Santos Cruxen C., Marques J.D.L., da Silva W.P., Fiorentini M. (2017). Essential oil from pink pepper as an antimicrobial component in cellulose acetate film: Potential for application as active packaging for sliced cheese. LWT-Food Sci. Technol..

[12] Gigante V., Aliotta L., Ascrizzi R., Pistelli L., Zinnai A., Batoni G., Coltelli M.-B., Lazzeri A. (2023). Innovative Biobased and Sustainable Polymer Packaging Solutions for Extending Bread Shelf Life: A Review. Polymers.

[13] Custom Market Insights Bread Market Size, Trends and Insights by Product Type (Bread & Buns, Cakes & Pastries), by Category (Conventional, Gluten-free), by Distributional Channel (Supermarket & Hypermarket, Bakery, Online), and by Region—Global Industry Overview, Statistical Data, Competitive Analysis, Share, Outlook, and Forecast 2023–2030. https://www.custommarketinsights.com/report/bread-market/.

[14] Diowksz A., Kopeć P., Koziróg A. (2024). The Inactivation of Microscopic Fungi in Bakery Products Using Hurdle Technology—A Case Study. Appl. Sci..

[15] Legan J.D. (1993). Mould spoilage of bread: The problem and some solutions. Int. Biodeterior. Biodegrad..

[16] Yazdi J.S., Salari M., Ehrampoush M.H., Bakouei M. (2024). Development of active chitosan film containing bacterial cellulose nanofibers and silver nanoparticles for bread packaging. Food Sci. Nutr..

[17] Garcia M.V., Bernardi A.O., Copetti M.V. (2019). The fungal problem in bread production: Insights of causes, consequences, and control methods. Curr. Opin. Food Sci..

[18] Císarová M., Hleba L., Medo J., Tančinová D., Mašková Z., Čuboň J., Kováčik A., Foltinová D., Božik M., Klouček P. (2019). The in vitro and in situ effect of selected essential oils in vapour phase against bread spoilage toxicogenic aspergilli. Food Control.

[19] Noshirvani N., Le Coz C., Gardrat C., Ghanbarzadeh B., Coma V. (2024). Active Polysaccharide-Based Films Incorporated with Essential Oils for Extending the Shelf Life of Sliced Soft Bread. Molecules.

[20] Sripahco T., Khruengsai S., Pripdeevech P. (2023). Biodegradable antifungal films from nanocellulose-gellan gum incorporated with Anethum graveolens essential oil for bread packaging. Int. J. Biol. Macromol..

[21] Safakas K., Giotopoulou I., Giannakopoulou A., Katerinopoulou K., Lainioti G.C., Stamatis H., Barkoula N.-M., Ladavos A. (2023). Designing Antioxidant and Antimicrobial Polyethylene Films with Bioactive Compounds/Clay Nanohybrids for Potential Packaging Applications. Molecules.

[22] (2024). Standard Test Method for Oxygen Gas Transmission Rate Through Plastic Film and Sheeting Using a Coulometric Sensor.

[23] Blois M.S. (1958). Antioxidant Determinations by the Use of a Stable Free Radical. Nature.

[24] Payum T., Das A.K., Shankar R., Tamuly C., Hazarika M. (2015). Antioxidant Potential of Solanum spirale Shoot and Berry: A Medicinal Food Plant Used in Arunachal Pradesh. Am. J. PharmTech Res..

[25] Bondet V., Brand-Williams W., Berset C. (1997). Kinetics and Mechanisms of Antioxidant Activity Using the DPPH• Free Radical Method. LWT-Food Sci. Technol..

[26] Bonilla J., Atarés L., Vargas M., Chiralt A. (2012). Edible films and coatings to prevent the detrimental effect of oxygen on food quality: Possibilities and limitations. J. Food Eng..

[27] Talegaonkar S., Sharma H., Pandey S., Mishra P.K., Wimmer R., Grumezescu A.M. (2017). 3-Bionanocomposites: Smart biodegradable packaging material for food preservation. Food Packaging: Nanotechnology in the Agri-Food Industry.

[28] Gupta P. (2023). Role of oxygen absorbers in food as packaging material, their characterization and applications. J. Food Sci. Technol..

[29] Hotta S., Paul D.R. (2004). Nanocomposites formed from linear low density polyethylene and organoclays. Polymer.

[30] Khalili S., Masoomi M., Bagheri R. (2012). The effect of organo-modified montmorillonite on mechanical and barrier properties of linear low-density polyethylene/low-density polyethylene blend films. J. Plast. Film Sheeting.

[31] Coelho L.B., Geraldine R.M., Silveira M.F.A., de Souza A.R.M., Torres M.C.L., Gonçalves M., Ássima B. (2020). Characterization of films of low density polyethylene incorporated with oregano essential oil. Res. Soc. Dev..

[32] Tornuk F., Sagdic O., Hancer M., Yetim H. (2018). Development of LLDPE based active nanocomposite films with nanoclays impregnated with volatile compounds. Food Res. Int..

[33] Ramos M., Beltr’an A., Peltzer M., Valente A.J.M., Garrig’os M.D.C. (2014). Release and antioxidant activity of carvacrol and thymol from polypropylene active packaging films. LWT-Food Sci. Technol..

[34] Pyrzynska K., Pekal A. (2013). Application of free radical diphenylpicrylhydrazyl (DPPH) to estimate the antioxidant capacity of food samples. Anal. Methods.

[35] Balaguer M.P., Lopez-Carballo G., Catala R., Gavara R., Hernandez-Munoz P. (2013). Antifungal properties of gliadin films incorporating cinnamaldehyde and application in active food packaging of bread and cheese spread foodstuffs. Int. J. Food Microbiol..

[36] Passarinho A.T.P., Dias N.F., Camilloto G.P., Cruz R.S., Otoni C.G., Moraes A.R.F., Soares N.d.F.F. (2014). Sliced bread preservation through oregano essential oil-containing sachet. J. Food Process Eng..

[37] Fonseca L.M., Souza E.J.D., Radünz M., Gandra E.A., da Rosa Zavareze E., Dias A.R.G. (2021). Suitability of starch/carvacrol nanofibers as biopreservatives for minimizing the fungal spoilage of bread. Carbohydr. Polym..

[38] Guarda A., Rubilar J.F., Miltz J., Galotto M.J. (2011). The antimicrobial activity of microencapsulated thymol and carvacrol. Int. J. Food Microbiol..

[39] Abbaszadeh S., Sharifzadeh A., Shokri H., Khosravi A., Abbaszadeh A. (2014). Antifungal efficacy of thymol, carvacrol, eugenol and menthol as alternative agents to control the growth of food-relevant fungi. J. Mycol. Medicale.

[40] Cheng M., Wang J., Zhang R., Kong R., Lu W., Wang X. (2019). Characterization and application of the microencapsulated carvacrol/sodium alginate films as food packaging materials. Int. J. Biol. Macromol..

[41] Jesus F.P.K., Ferreiro L., Bizzi K.S., Loreto É.S., Pilotto M.B., Ludwig A., Alves S.H., Zanette R.A., Santurio J.M. (2015). In vitro activity of carvacrol and thymol combined with antifungals or antibacterials against Pythium insidiosum. J. Mycol. Med..

[42] Ramos M., Beltrán A., Valdes A., Peltzer M.A., Jiménez A., Garrigós M.C., Zaikov G.E. (2013). Carvacrol and thymol for fresh food packaging. J. Bioequiv. Bioavailab..

[43] Schlemmer K.B., Jesus F.P.K., Tondolo J.S.M., Weiblen C., Azevedo M.I., Machado V.S., Botton S.A., Alves S.H., Santurio J.M. (2019). In vitro activity of carvacrol, cinnamaldehyde and thymol combined with antifungals against Malassezia pachydermatis. J. Mycol. Med..

[44] Saranraj P., Geetha M. (2012). Microbial Spoilage of Bakery Products and Its Control by Preservatives. Int. J. Pharm. Biol. Arch..

[45] Gavaric N., Mozina S.S., Kladar N., Bozin B. (2015). Chemical Profile, Antioxidant and Antibacterial Activity of Thyme and Oregano Essential Oils, Thymol and Carvacrol and Their Possible Synergism. J. Essent. Oil-Bear. Plants.

[46] Klinmalai P., Srisa A., Laorenza Y., Katekhong W., Harnkarnsujarit N. (2021). Antifungal and plasticization effects of carvacrol in biodegradable poly(lactic acid) and poly(butylene adipate terephthalate) blend films for bakery packaging. LWT.

[47] Suwanamornlert P., Kerddonfag N., Sane A., Chinsirikul W., Zhou W., Chonhenchob V. (2020). Poly(lactic acid)/poly(butylene-succinate-co-adipate) (PLA/PBSA) blend films containing thymol as alternative to synthetic preservatives for active packaging of bread. Food Packag. Shelf Life.

[48] Canales D., Montoille L., Rivas L.M., Ortiz J.A., Yañez-S M., Rabagliati F.M., Ulloa M.T., Alvarez E., Zapata P.A. (2019). Fungicides Films of Low-Density Polyethylene (LDPE)/Inclusion Complexes (Carvacrol and Cinnamaldehyde) Against Botrytis Cinerea. Coatings.

[49] Campos-Requena V.H., Rivas B.L., Pérez M.A., Figueroa C.R., Sanfuentes E.A. (2015). The synergistic antimicrobial effect of carvacrol and thymol in clay/polymer nanocomposite films over strawberry gray mold. LWT-Food Sci. Technol..

[50] Cid-Pérez T.S., Munguía-Pérez R., Nevárez-Moorillón G.V., Ochoa-Velasco C.E., Navarro-Cruz A.R., Avila-Sosa R. (2024). Carvacrol and thymol effect in vapor phase on *Escherichia coli* and *Salmonella* serovar Typhimurium growth inoculated in a fresh salad. Heliyon.

[51] Shemesh R., Krepker M., Natan M., Danin-Poleg Y., Banin E., Kashi Y., Nitzan N., Vaxman A., Segal E. (2015). Novel LDPE/halloysite nanotube films with sustained carvacrol release for broad-spectrum antimicrobial activity. RSC Adv..

[52] Devecioglu D., Turker M., Karbancioglu-Guler F. (2022). Antifungal Activities of Different Essential Oils and Their Electrospun Nanofibers against Aspergillus and Penicillium Species Isolated from Bread. ACS Omega.

[53] Fernandes F.G., Grisi C.V.B., Costa Araújo R., Botrel D.A., Sousa S. (2021). Active cellulose acetate-oregano essential oil films to conservation of hamburger buns: Antifungal, analysed sensorial and mechanical properties. Packag. Technol. Sci..

[54] Heras-Mozos R., Muriel-Galet V., López-Carballo G., Catalá R., Hernández-Muñoz P., Gavara R. (2019). Development and optimization of antifungal packaging for sliced pan loaf based on garlic as active agent and bread aroma as aroma corrector. Int. J. Food Microbiol..

